# Novel cancerization marker, *TP53*, and its role in distinguishing normal tissue adjacent to cancerous tissue from normal tissue adjacent to benign tissue

**DOI:** 10.1186/1477-7819-10-252

**Published:** 2012-11-21

**Authors:** Guo-Yan Liu, Kun-Hong Liu, Yin Li, Chao Pan, Ji-Qin Su, Hong-Feng Liao, Ren-Xiang Yv, Zhao-Hui Li, Li Yuan, Huan-Jing Zhang, Chi-Meng Tzeng, Bing Xiong

**Affiliations:** 1 Department of Oncology, Zhongnan Hospital of Wuhan University, Wuhan, Hubei, 430071, PR China; 2 Hubei Cancer Clinical Study Center, Wuhan, Hubei, 430071, PR China; 3 Hubei Key Laboratory of Tumor Biological Behaviors, Wuhan, Hubei, 430071, PR China; 4 Zhongshan Hospital Affiliated to Xiamen University, Xiamen, 361004, PR China; 5 Software School of Xiamen University, Xiamen, 361005, PR China; 6 Xiamen Entry-Exit Inspection and Quarantine Bureau, Xiamen, 361005, PR China; 7 School of Life Sciences, Key Laboratory of the Ministry of Education for Cell Biology and Tumor Cell Engineering, Xiamen University, Xiamen, 361005, PR China; 8 College of Pharmaceutical Sciences, Translational Medicine Research Center, Xiamen University, Xiamen, 361102, PR China

**Keywords:** Cancerization, Genetic biomarkers, Normal tissue adjacent to benign, Normal tissue adjacent to cancer, Tissue microarray

## Abstract

**Background:**

The histopathological and molecular heterogeneity of normal tissue adjacent to cancerous tissue (NTAC) and normal tissue adjacent to benign tissue (NTAB), and the availability of limited specimens make deciphering the mechanisms of carcinogenesis challenging. Our goal was to identify histogenetic biomarkers that could be reliably used to define a transforming fingerprint using RNA *in situ* hybridization.

**Methods:**

We evaluated 15 tumor-related RNA *in situ* hybridization biomarkers using tumor microarray and samples of seven tumor-adjacent normal tissues from 314 patients. Biomarkers were determined using comprehensive statistical methods (significance of support vector machine-based artificial intelligence and area under curve scoring of classification distribution).

**Results:**

*TP53* was found to be a most reliable index (*P* <10^-7^; area under curve >87%) for distinguishing NTAC from NTAB, according to the results of a significance panel (*BCL10*, *BECN1*, *BRCA2*, *FITH*, *PTCH11* and *TP53*).

**Conclusions:**

The genetic alterations in *TP53* between NTAC and NTAB may provide new insight into the field of cancerization and tumor transformation.

## Background

At present, tumor-adjacent tissue samples are considered as normal specimens and normal controls in histopathological applications, and therefore often used as a standard negative control to determine whether malignant tumors have been removed cleanly [1]. However, we cannot guarantee that normal tissue adjacent to cancerous tissue (NTAC) has been unaffected by the nearby malignant tumor. Normal tissue adjacent to benign tissue (NTAB) has been shown to be histologically and genetically normal, but the issue of distinguishing one from the other in a reliable manner has continued to elude researchers.

A growing tumor body surrounded by pathologist-validated NTAC is by definition abnormal at the molecular level because of long-term expansion or clonal conversion from patch to field of cancerization [1-4]. Those two models have been implicated mainly in malignant tumors of the breast, skin, prostate, lung, liver, brain and gastrointestinal tract [4-9]. However, wound-healing does not occur in tissues adjacent to invasive cancers [10]. The epithelial-mesenchymal transition that initiates the invasion process of most tumors has also been observed in NTAC [11,12]. Moreover, diverse genetic studies of field cancerization have assessed the copy number, expression and single nucleotide polymorphisms in the genomic DNA and messenger RNA of NTAC, but they cannot explain the mechanisms behind tumor progression, metastasis or recurrence [2,13,14]. The genetic alterations between NTAC and NTAB may provide new insight into the field of cancerization and tumor transformation.

Tumor development is a smooth process that goes through several molecular stages, including gene transformation [15,16]. Tumor cells interact with adjacent normal cells, indicating gene cross-talk and mutual signal transduction from the two kinds of cells [15,17-19]. Relative to benign tumor cells, malignant tumor cells show more aggressive cellular growth and integration. Thus, we could expect that benign and malignant tumors and their adjacent tissues would undergo different malignant transformations.

The genes that initiate tumor processes are collectively known as tumor-related genes (TRGs) and comprise oncogenes, tumor-suppressor genes, and genes that promote and inhibit cancer progression and metastasis. To date, many TRGs from malignant tumors and other cells have been identified and intensively studied for the purposes of explaining the molecular mechanism of cancer development, drug discovery and diagnostics [16,20,21]. Nevertheless, few clinical studies have been specifically devoted to the rule of TRGs in distinguishing NTAC from NTAB.

In this study, we collected tumors that were malignant (six types) and benign (two types) and adjacent tissue samples (eight types) from 314 patients to generate a tumor microarray (TMA). We then used 15 histogenetic cancer markers, or TRGs (*MYC* (ENSG00000136997), *CCND1* (ENSG00000110092*)*, *TP53* (ENSG00000141510*)*, *UVRAG* (ENSG00000171862*)*, *RB1* (ENSG00000139687*)*, *PTEN* (ENSG00000171862), *PTCH1* (ENSG00000185920*)*, *BRCA1* (ENSG00000012048*)*, *BRCA2* (ENSG00000139618), *FHIT* (ENSG00000189283*)*, *BECN1* (ENSG00000126581*)*, *BCL10* (ENSG 00000142867), *APC* (ENSG00000134982*)*, *CD82* (ENSG00000085117) and *NME1*-*NME2* (ENSG 00000011052)) for finding novel biomarkers involved in cancerization or tumor transformation or recurrence through RNA *in situ* hybridization (RISH) and comprehensive statistical analysis.

## Methods

### Specimen collection and tissue microarray composition

We collected 314 primary tumor biopsy samples from Chinese patients at Zhongshan Hospital, which is affiliated with Xiamen University. Written informed consent was obtained from the patients for publication of this report and any accompanying images. The specimens were collected from 2000 to 2006. Samples of normal tissue adjacent to tumor samples were flash-frozen and stored at −70°C before further treatment. Tumors included hepatocellular carcinoma (26 cases), rectal adenocarcinoma (48 cases), esophageal squamous cell carcinoma (34 cases), gastric adenocarcinoma (66 cases), thyroid carcinoma (32 cases), breast carcinoma (38 cases), thyroid adenoma (32 cases) and breast fibroadenoma (38 cases). Histologically normal tissues adjacent to tumors were selected from the incised edges of the resected tumors. Tissue blocks measuring approximately 1.5 × 1.5 × 0.3 cm were fixed in PBS containing 4% paraformaldehyde (1% diethyl pyrocarbonate, pH 7.4) for 24 hours at 4°C. Standard treatment for paraffin sections under an RNase-free control condition was then performed. Sections stained with hematoxylin and eosin were reviewed under microscopes to confirm the presence of histologically normal or cancerous areas. Duplicated TMA chips had 1-mm-diameter TMA cores with 0.8 mm of space between the core centers. We generated two sets of TMA of tumors (malignant and benign) and para-tissue (NTAC and NTAB) for the following RISH examination.

### Preparation of tumor marker probes

Through an article search of the National Center for Biotechnology Information PubMed database and the most common-use RISH commercial kits (Cybrdi, Rockville, MD, USA, we selected 15 TRGs as a starting screening panel. Antisense probes, perfectly matched to each corresponding sequence, were prepared using a ‘locked nucleic acid’ (LNA) modification (ribose ring of the nucleotide ‘locked’ with a methylene bridge connecting the 2′-O atom with the 4′-C atom) to increase stability and sensitivity. Probes information is shown below:

(* indicates LNA modifications)

**APC** 5-TTGGTTCCCAGATGACTTGTCAGCCT*TCG AGGTGCAGAGTGTGTG CTACTAG-3dig;

**BCL10** 5-CTGTATCAGGAAGTTCTGTGT*TTTTTCTCGCCGAATAGATTCAACAAGGGTG-3dig,

**BECN1** 5-CCAAGCAGCATTAATCTCATTCCAT*TCCACGGGAACACTGGGCAGGCGACC-3dig;

**BRCA1** 5-CCTCTTTCTTCATCATCTGAAACCAATT*CCTTGTCACTCAGACCAACTCCCT-3dig;

**BRCA2** 5-AAGCGATGATAAGGGCAGAGGAAAAGGT*CTAGGGTCAGGAAAGAATCCAAGT-3dig;

**FHIT** 5-AGTCCTCCTTGTCATGTTTCTGGAGCT*CCTCATAGATGCTGTCATTCCTGTG-3dig;

**CD82** 5-GCAGAAGCCCTTCCTCACAGAAAGGCT*GTTGTCCTCTTCCCCCTTGACTTCGC-3dig;

**NME1** 5-GGAATCCTTTCTGCTCAAAACGCT*TGATAATCTCTCCCACAAGACCCCGCTG-3dig;

**RB1** 5-TGAGCACACGGTCGCTGTTACAT*ACCATCTGATTTATTTTCTGGAACTTCT-3dig;

**PTEN** 5-CCTCTTGATATCTCCTTTTGTTTCT*GCTAACGATCTCTTTGATGATGGCTG-3dig;

**PTCH1** 5-CGCTTCTGTGGTCAGGACATT*AGCACCTTCTTCTTTAGGGGTCTGTATCAT-3dig;

**UVRAG** 5-CTCCTTGTTCTTGGCTAGGGTGCACAT*TCGCGTGGCCTCCGTTTAAGCTGCCAAC-3dig;

**TP53** 5-CCAGGACAGGCACAAACACGCACCT*CAAAGCTGTTCCGTCCCAGTAGATTAC-3dig;

**CCND1** 5-CCTCCTCGCACTTCTGTTCCTCGCAGACCT*CCAGCATCCAGGTGGCGACGATCTTCCG-3dig;

**MYC** 5-CTTCCTCATCTTCTTGTTCCTCCTCAGAGT*CGCTGCTGGTGGTGGGCGGTGTC-3dig.

### RNA *in situ* hybridization and quantification

The hybridization procedures performed in this study were performed in accordance with the RISH kit manufacturer’s instructions (Cybrdi) with several modifications: vanadyl- ribonucleoside complex (1 mM) was added to keep RNase from causing RNA degradation, and cetyltrimethylammonium bromide was used to structurally stabilize the hybridization between oligo-probes and complimentary targets. LNA was used to improve the stability and sensitivity of the monomer probes. (Detailed protocol available upon request.) We optimized RISH with 10 ng/μL probe concentration, onto tissue microarray chip (TMC) with regards digestion (min) and incubation (h) time, incubation temperature (°C) and chromogenic time (min), respectively (Table 1). Of the TRGs, *MYC* was found to be 20 min / 42 h / 41.5°C / 30 min, *CCND1* was found to be 20 min / 36 h / 45°C / 50 min, *TP53* was found to be 30 min / 44 h / 48°C / 110 min, *UVRAG* was found to be 30 min / 38 h / 18.5°C / 60 min, *RB1* was found to be 25 min / 42 h / 21°C / 45 min, *PTEN* was found to be 20 min / 40 h / 19.5°C / 45 min, *PTCH1* was found to be 22 min / 40 h / 23°C / 40 min, *BRCA1* was found to be 24 min / 39 h / 22°C / 40 min, *BRCA2* was found to be 24 min / 39 h / 23°C / 35 min, *FHIT* was found to be 20 min / 44 h / 24°C / 40 min, *BECN1* was found to be 24 min / 46 h / 20.5°C / 25 min, *BCL10* was found to be 25 min / 40 h / 19.5°C / 90 min, *APC* was found to be 25 min / 37 h / 20°C / 80 min, *CD82* was found to be *25* min / 40 h / 29°C / 35 min, and *NME1-NME2* was found to be 22 min / 46 h / 20.5°C / 50 min*.*

**Table 1:** **Optimal conditions for RNA *in situ *hybridization**

**Tumor-related gene**	**Digestion time (min)**	**Incubation time (h)**	**Incubation temperature (°C)**	**Chromogenic time (min)**
*MYC*	20	42	41.5	30
*CCND1*	20	36	45	50
*TP53*	30	44	48	110
*UVRAG*	30	38	18.5	60
*RB1*	25	42	21	45
*PTEN*	20	40	19.5	45
*PTCH1*	22	40	23	40
*BRCA1*	24	39	22	40
*BRCA2*	24	39	23	35
*FHIT*	20	44	24	40
*BECN1*	24	46	20.5	25
*BCL10*	25	40	19.5	90
*APC*	25	37	20	80
*CD82*	25	40	29	35
*NME1-NME2*	22	46	20.5	50

RISH results were determined by measuring the ratio of positive cells to total cells and density of staining. The criteria for the positive cell ratio are scored as 0 for <25%, 1 for 25% to 50%, 2 for 51% to 75%, and 3 for >75%. Staining density was scored as 0 for no staining, 1 for light staining, 2 for deep staining, and 3 for black staining. Expression levels were scored from 0 to 6 using the sums of these two scores.

### Gene expression and statistical analysis

Two techniques for data analysis were implemented: a statistical method (analyses were performed using the SPSS 10.0 (SPSS Inc., Chicago, IL, USA) used to calculate the *P*-values (significance <0.05) of genes in different samples, and a support vector machine (SVM)-learning method applied to further discover the relationship between genes and corresponding samples.

The significance levels of the 15 TRGs were analyzed by Wilcoxon rank-sum test, which is an efficient nonparametric statistical method to compare two groups (NTAC and NTAB) of data and determine their differences. It is important to choose an efficient machine-learning method to further explore the connections between genes and different cancers. However, it is hard to decide what kind of functions the 15 TRGs would have for the different types of cases. So it is necessary to separately analyze the effects of both a single gene and different gene groups in different specimens. However, since there are so many ways to construct a gene group within 15 genes, efficient methods are required to shrink the scope of gene group construction.

To achieve this, four classical feature selection methods were used to analyze gene expression levels, including: *t* test, entropy, Bhattacharyya and Wilcoxon. All these methods were provided in a bioinformatics toolbox embedded in MATLAB 7.1 (MathWorks, Inc., Natick, MA, USA ). Based on different criteria for feature selection, different methods would result in genes in different order of importance. The genes were classified into different groups. The discriminatory ability of the gene groups was measured by SVM. This is a supervised learning model with associated learning algorithm that analyzes data and recognizes patterns, used for classification and regression analysis. Comparing the results revealed the genes with biological significance.

There were three steps to analyze gene expressions: firstly, the gene expressions of different specimens were measured with the Wilcoxon rank-sum test, so that the *P*-value of each gene could be calculated, and then used to evaluate the homological extent of the two specimens. Secondly, the classification ability of each gene was analyzed singly among different tissues to further assess the importance of each gene in different tissues. Thirdly, the results obtained by the combination of different genes were investigated. The relationship among genes could be discovered in this way. It is easy to directly evaluate the classification ability of a single gene using SVM with 10-fold cross-validation (CV). However, because there are many ways to select the 15 genes to form a gene group, it is necessary to take a reliable selection method. In our analysis, we started with an empty gene. A filter method was applied to rank the genes, and then a gene was added to the group according to the score of the rank. The gene group was used to discriminate the samples in two types of tissues using SVM by the 10-fold CV method. This process ended when all genes were added to the group. In addition, as the sample sizes varied in different diseases, the area under the curve (AUC) of the receiver-operating characteristic was deployed in our experiments. A the receiver-operating characteristic curve represents the true positive rate as a function of the corresponding false positive rate, and the AUC provides a measure of performance that is sensitive to the distribution of the activity classes in test sets. Using the AUC, the problem of sample size unbalance can be solved and the best gene subset can be determined. Lastly, the best gene subsets can be found by the highest AUC.

## Results

Pathologists identified malignant and benign tumors through immunohistocompatibility observation and by RISH onto Formalin-Fixed Paraffin-Embedded (FFPE) slides or TMA. From immunohistocompatibility microscopic observation, we histologically confirmed eight major carcinomas, benign tumors and associated NTAC or NTAB tissue.

**Figure 1:**
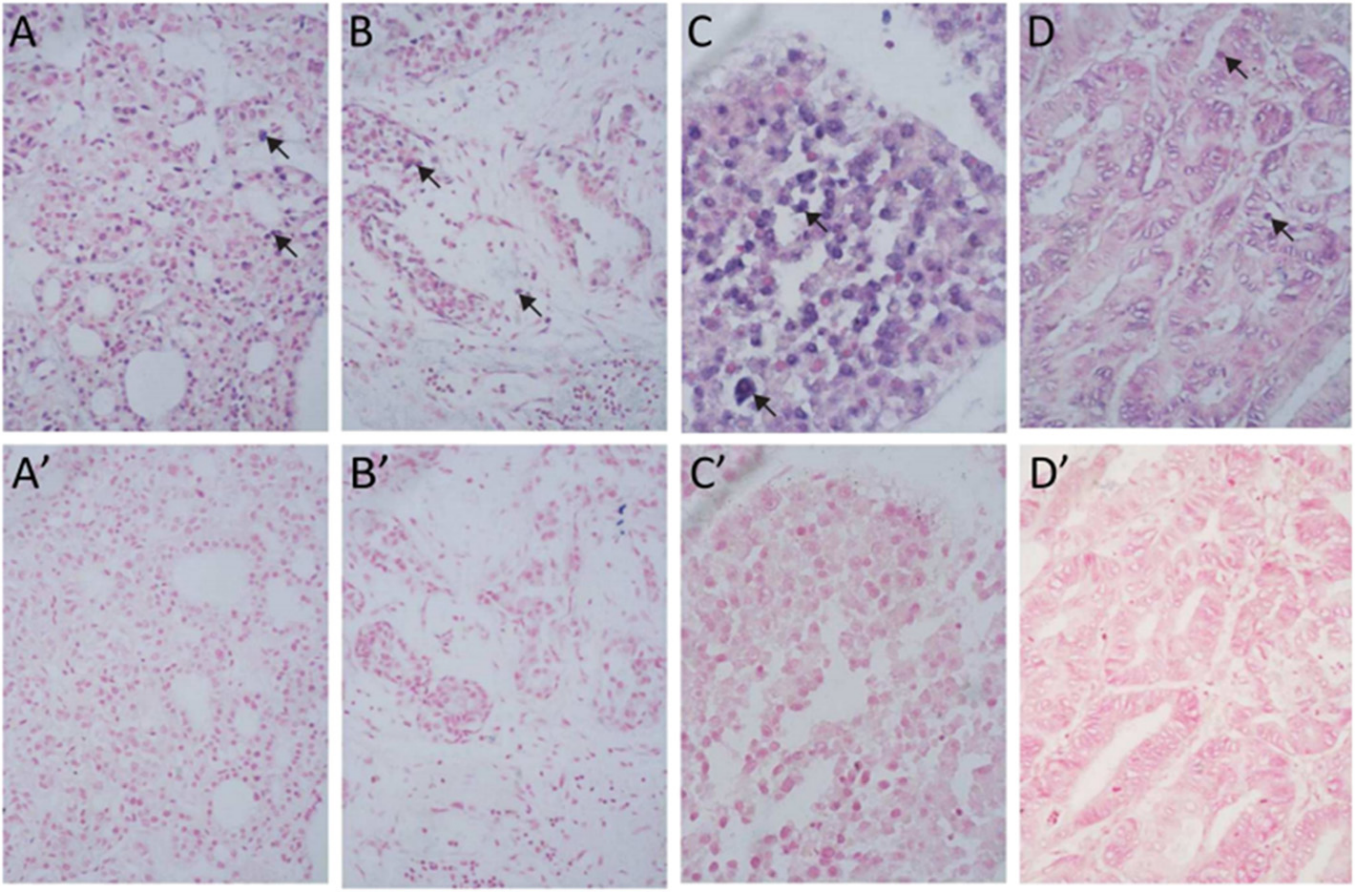
**RNA *in situ *hybridization of TP53 expression for normal tissue adjacent to cancerous tissue and normal tissue adjacent to benign tissue.** (**A**-**D**) show the NTAC from the thyroid, breast, liver and colon cancer samples, respectively. (**A**′-**D**′) show the NTAB. Unlike in malignant RISH, there is no clonal patch, but there is a scattered TP53-positive cell distribution, indicated by the arrow. The positive cell number and density were visualized on a Zeiss-Axiophot DM HT microscope (Zeiss-Axiophot, Oberkochen, Germany) and captured with an attached camera linked to a computer plus EZ-HYB software. NTAB: normal tissue adjacent to benign tissue; NTAC: normal tissue adjacent to cancerous tissue; RISH: RNA *in situ* hybridization.

Deciphering RISH through malignant biopsies, we found that all histological RISH patterns were similar to those discerned during prior clinical observation. The clusters were highly dense, and there were patches of positive cells (data not shown). Most NTACs and NTABs were arrayed on TMA. They also showed a strong distribution of cells positive for 15 tumor marker probes. The scattered distribution of probes were calculated and further scored the gene expression level of each of the 15 genetic markers (*APC*, *BCL10*, *BECN1*, *BRCA1*, *BRCA2*, *FHIT*, *CD82*, *NME1*, *RB1*, *PTEN*, *PTCH1*, *UVRAG*, *TP53*, *CCND1*, *MYC*) by positive cell count and measurement of staining density. Figure 1 shows the RISH of TP53 expression for NTAC and NTAB in thyroid, breast and liver tissue, and in benign colon cancer.

No significant difference in expression was found between malignant tumors, between benign tumors, or between their related adjacent tissue. However, we did find differences in expression between NTAC and NTAB. Ten overexpressed and one underexpressed TRGs were identified: *BECN1* with *P <*10^-8^, *BCL10* with *P <*10^-7^, *BRCA1* with *P <*10^-4^, *BRCA2* with *P <*10^-12^, *FITH* with *P <*10^-10^, CD82* with *P <*10^-5^, *PTCH11* with *P <*10^-9^, *PTEN* with *P <*10^-4^, *TP53* with *P <*10^-12^, *CyclinD1* with *P <*10^-5^, and *MYC* with *P <*10^-5^.

**Figure 2:**
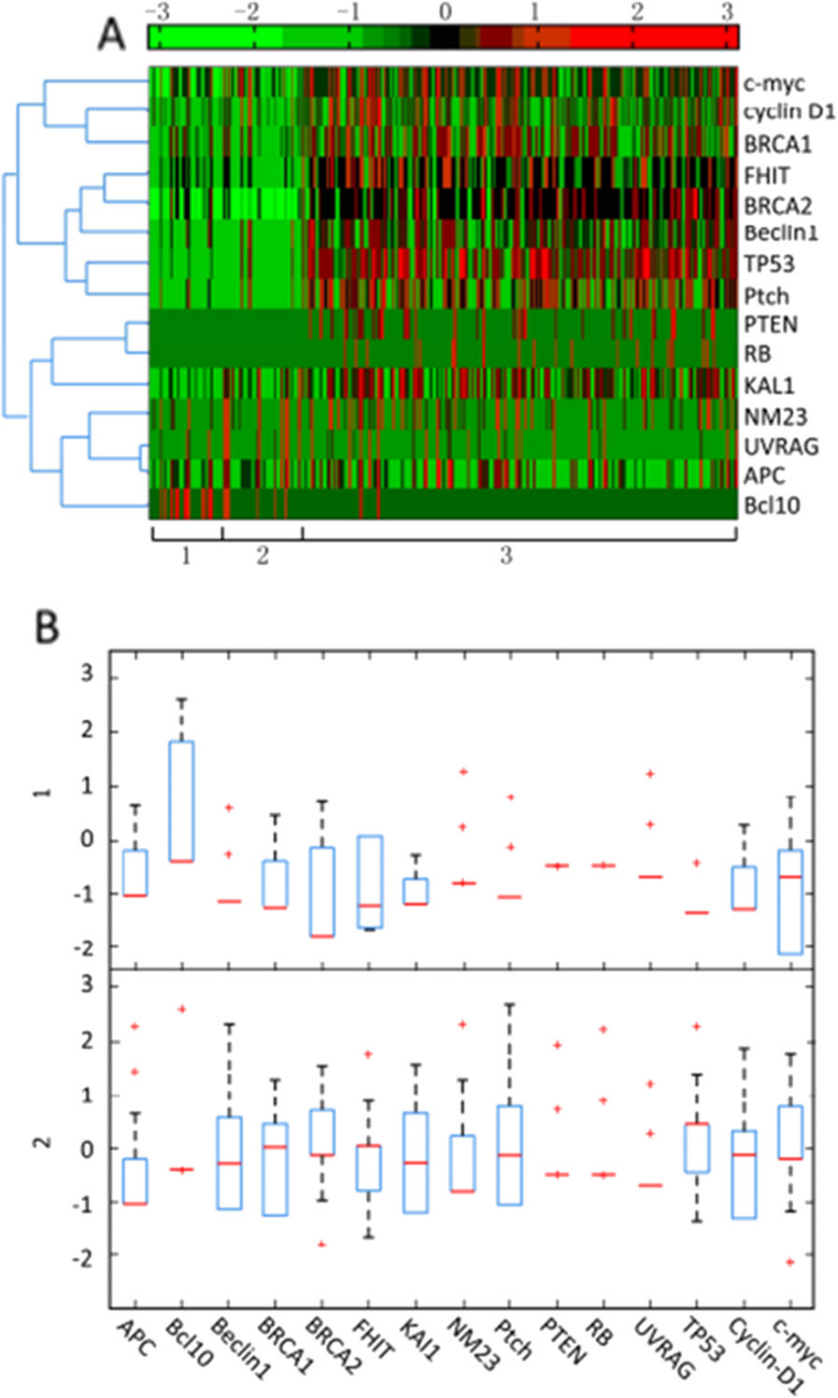
**RNA *in situ *hybridization expression profile of 15 tumor-related genes between normal tissue adjacent to cancerous tissue and normal tissue adjacent to benign tissue from 314 patients.** (**A**) Heat map and tree map of RISH expression for 15 TRGs. (1) and (2) NTAB in thyroid and breast tissue. (3) NTAC for all malignant tumors. Units −3 through +3 represents the scale of gene expression corresponding to the sample. (**B**) Box plot distribution of TRG expression in (1) NTAB and (2) NTAC for 314 biopsies. NTAB: normal tissue adjacent to benign tissue; NTAC: normal tissue adjacent to cancerous tissue; RISH: RNA *in situ* hybridization; TRG: tumor-related gene.

**Figure 3:**
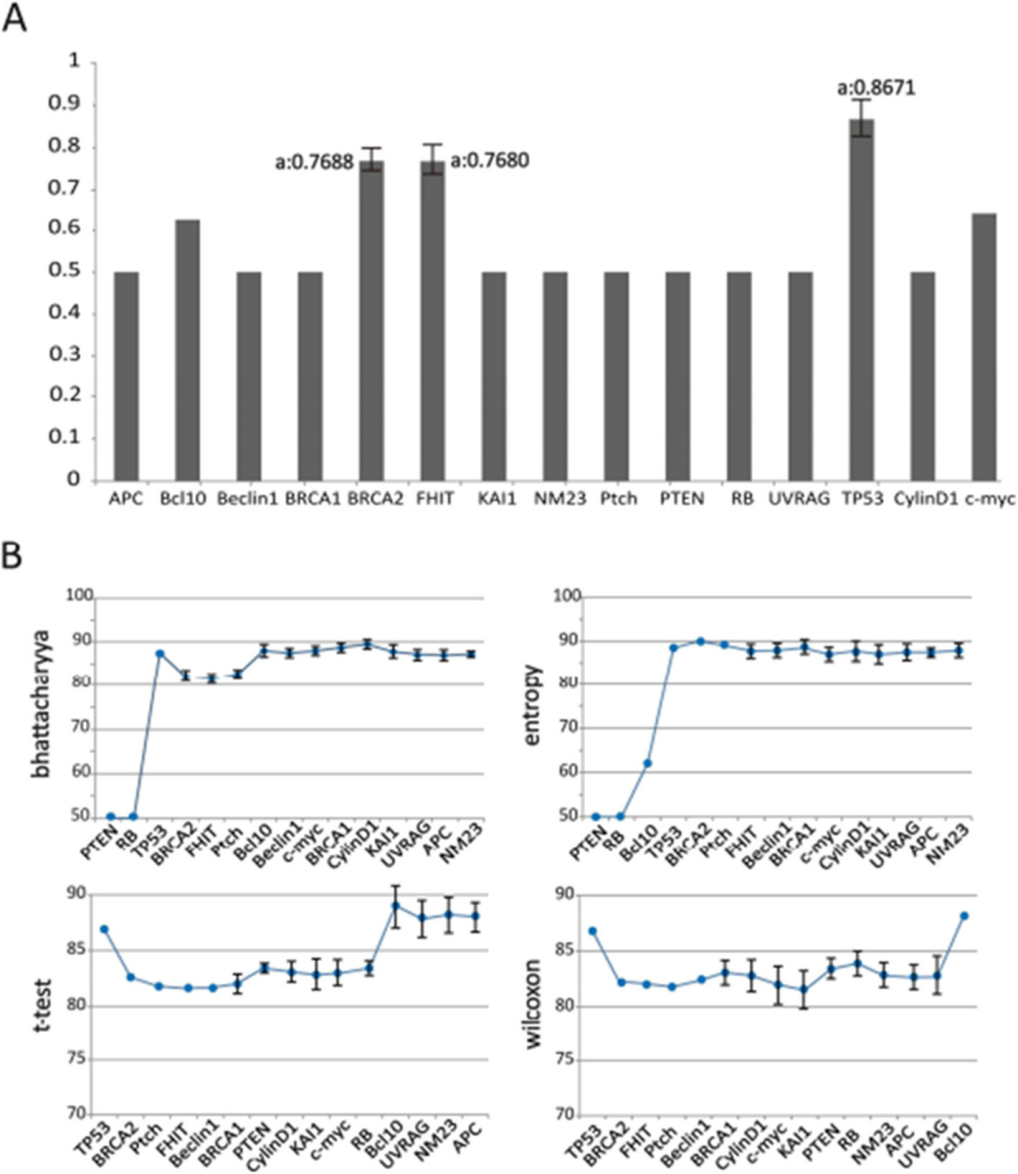
**The result of 15 tumor-related genes analysis.** (**A**) AUC values from 15 tumor-related genes in normal tissue adjacent to cancerous tissue and normal tissue adjacent to benign tissue groups. X-axis: 15 tumor-related genes; Y-axis: AUC values. (**B**) Results of ten runs of 10-fold CV for gene expression in NTAB versus NTAC. Each figure represents the of one filter method. X-axis: orders of selected genes. Y-axis: average and standard deviation of the AUC scores obtained in the ten 10-fold CV with the corresponding gene groups. AUC: area under the curve; CV: cross-validation; NTAB: normal tissue adjacent to benign tissue; NTAC: normal tissue adjacent to cancerous tissue.

Through analysis of the NTAC heat map, *c-Myc*, *CyclinD1*, *BRAC1*, *FITH*, *BRCA2*, *PTEN*, *TP53* and *PTCH1* were found to cause positive fold changes relative to NTAB (Figure 2A). In parallel, through analysis of the box plot of the distribution of gene expression in NTAC and NTAB, *BRCA1*, *BRCA2*, *TP53* and *CyclinD1* showed significant differences (Figure 2B). Meanwhile, we used AUC measurement and four classical statistical methods to filter ranking order for these 15 genes. We used 10-fold CV runs, *t*-testing, entropy, Brattacharyya distance and Wilcoxon rank-sum testing to smooth out discrepancies between the heat map and box plot (Figure 3).

Brattacharyya distance and entropy showed *PTEN*, *TP53* and *RB1* to be the most significant genes, but *PTEN* and *RB1* showed lower AUC values, below 50%. *TP53* was found to possess over 87% AUC to be the most reliable biomarker for distinguishing NTAC and NTAB.

In *t*- and Wilcoxon rank-sum tests, *TP53* was found to be the best indicator, with an AUC value of 86%. This implies that *TP53* plays a critical role in cell transformation or cancerization.

## Discussion

We selected 15 TRGs known to act on various aspects of tumor development. These included genes involved in apoptosis (*TP53*, *BCL10*), cell-cycle control (*RB1*/*RB*, *APC*, *CCND1*), DNA repair (*BRCA1*, *BRCA2*), autophagy regulation (*UVRAG*, *BECN1*), signaling and enzyme activity (*PTEN*, *PTCH1*, *FHIT*), and metastasis (*NME1–NME2*, *CD82*) and a single oncogene (*MYC*). These genes were frequently reported as significantly different in expression level between selected malignant tumors and adjacent normal tissue. However, our RISH results showed that, between NTAC and NTAB, the expressions of 11 of these 15 genes were significantly different, implying that NTAC and NTAB (baseline) share unlikely tissues and molecular patterns, even though, immunohistocompatibility shows them to be identical. It can be concluded that NTAC is subject to cancer regulation, transformation, and even cancerization, and cannot be defined as a tumor-free baseline or negative control. Consequently, TRGs could be considered molecular indexes for monitoring the transformation of cancer transformation from normal tissues, which is a better means for cancer prognosis than a histological method.

Related clonality and independent multiple lesions are two major hypotheses of field cancerization, but they cannot explain the genetic alterations observed in NTAC. We found no clonal patch or loci of independent lesions or clusters. Instead, we found a scattered distribution of positive cells. This could be explained by inducible field cancerization starting from the adjacent normal cells and spreading through molecular inducers of malignancy. Recently, scientists have proposed that tumor development involves a unique micro-environment that relies heavily on the neighborhood fibroblasts, endothelial cells and infiltrating fibroblasts. Inflammatory cells and immune cells infiltrate into nearby non-tumor cells and then transform the area into tumor territory [17,22].

Despite this, more than 10 TRGs were found to be significantly expressed in NTAC compared with NTAB. We customized the SVM-learning method to minimize the empirical classification error and maximize the geometric margin. This mainly nullified TRGs but it also nullified *TP53* from seven tumor types. In malignant tumor research, *TP53* is the most notable dysfunctional suppressor in carcinogenesis. It behaves as a tumor marker of field cancerization in breast cancer, lung cancer, brain tumors, and skin cancer to hepatocellular carcinomas [4,23-30]. *TP53* was reported to be a valuable part of risk assessment and a prognostic biomarker of breast cancer, showing a high hazard ratio and statistical significance (*P* < 0.0001) [26].

## Conclusions

*TP53* has been applied as a diagnostic biomarker either by immunostaining or by genetic detection. We believed that field cancerization and tumor transformation are strongly related to NTAB or NTAC. Our finding, that *TP53* is a reliable index suitable for distinguishing NTAC from NTAB in many clinical biopsies, is going to benefit prognostics in malignant cancer monitoring and further prevention

At the same time, we are working on applying genome-wide association studies, miRNA and epigenetic methylation detection to fully decipher the molecular mechanism of cancerization and tumor formation.

## Abbreviations

AUC: Area under the curve; CV: Cross-validation; LNA: Locked nucleic acid; NTAB: Normal tissue adjacent to benign tissue; NTAC: Normal tissue adjacent to cancerous tissue; PBS: Phosphate-buffered saline; RISH: RNA *in situ* hybridization; SVM: Support vector machine; TMA: Cancerization tissue microarray; TRGs: Tumor-related genes; TMC: Tissue microarray chip; FFPE: Formalin-Fixed Paraffin-Embedded.

## Competing interests

The authors declare that they have no competing interests.

## Authors' contributions

GYL was the main initiator of this work; KHL carried out the bioinformatic analysis; YL prepared and generated TMA; CP and HFL completed the pathological diagnosis and classification; JQS, ZHL and RXY performed clinical and empirical data recoding and analysis; LY and HJZ were the principal investigators. Clinical advice and supporting, plus FFPE as tissue microarray were fully corresponding by MD, Bing Xiong. For the RNA probing design, RISH and PCR validation, Dr. Tzeng took all responsibility, plus English writing and editing. Those two people shared corresponding author as "co-coresponding". All authors read and approved the final manuscript.
